# Molecular Network-Based Identification of Tramadol Metabolites in a Fatal Tramadol Poisoning

**DOI:** 10.3390/metabo12070665

**Published:** 2022-07-19

**Authors:** Romain Magny, Nicolas Auzeil, Bertrand Lefrère, Bruno Mégarbane, Pascal Houzé, Laurence Labat

**Affiliations:** 1 Laboratoire de Toxicologie, Fédération de Toxicologie, AP-HP, Hôpital Lariboisière, 75006 Paris, France; bertrand.lefrere@aphp.fr (B.L.); pascal.houze@aphp.fr (P.H.); laurence.labat@aphp.fr (L.L.); 2 Université Paris Cité, CNRS, CiTCoM, 75006 Paris, France; nicolas.auzeil@parisdescartes.fr; 3 Réanimation Médicale et Toxicologique, Fédération de Toxicologie, AP-HP, Hôpital Lariboisière, 75010 Paris, France; bruno.megarbane@aphp.fr; 4 Inserm, UMRS-1144, Université Paris Cité, 75006 Paris, France; 5 Université Paris Cité, CNRS, INSERM, Unité des Technologies Chimiques Et Biologiques Pour La Santé (UTCBS), 75006 Paris, France

**Keywords:** high-resolution tandem mass spectrometry, untargeted metabolomics, clinical toxicology, molecular network, tramadol

## Abstract

Identification of xenobiotics and their phase I/II metabolites in poisoned patients remains challenging. Systematic approaches using bioinformatic tools are needed to detect all compounds as exhaustively as possible. Here, we aimed to assess an analytical workflow using liquid chromatography coupled to high-resolution mass spectrometry with data processing based on a molecular network to identify tramadol metabolites in urine and plasma in poisoned patients. The generated molecular network from liquid chromatography coupled to high-resolution tandem mass spectrometry data acquired in both positive and negative ion modes allowed for the identification of 25 tramadol metabolites in urine and plasma, including four methylated metabolites that have not been previously reported in humans or in vitro models. While positive ion mode is reliable for generating a network of tramadol metabolites displaying a dimethylamino radical in their structure, negative ion mode was useful to cluster phase II metabolites. In conclusion, the combined use of molecular networks in positive and negative ion modes is a suitable and robust tool to identify a broad range of metabolites in poisoned patients, as shown in a fatal tramadol-poisoned patient.

## 1. Introduction

Over the last decade, untargeted metabolomic analyses have been proven to be essential for the identification of unknown metabolites and to assess quantitative changes between samples obtained during the course of a pathological event [1,2,3]. Numerous metabolomic analyses are based on liquid chromatography coupled to high-resolution tandem mass spectrometry (LC-HRMS/MS) [1,2,3]. LC-HRMS/MS is a powerful tool to obtain reliable data regarding structural information and a valuable tool for quantification [4]. In untargeted metabolomic analyses, data-dependent acquisition (DDA) mode was introduced several years ago and facilitated metabolite identification in complex samples [4,5]. DDA corresponds to a mode of data acquisition in tandem mass spectrometry in which a defined number of precursor ions whose *m*/*z* values were recorded in a survey scan are selected and subjected to an additional mass selection in a MS/MS analysis [6]. These approaches allowed the covering of an important part of the metabolome in a given sample. Nevertheless, efficient, reliable, and comprehensive identification of the tremendous number of metabolites detected by mass spectrometers remains challenging.

Molecular networks (MNs) have been recently developed and used to identify new compounds, especially in medicinal chemistry, with their first historical applications in studies focused on metabolites obtained from plants [7,8]. MNs are a computational strategy of organizing and visualizing hundreds of molecules based on their MS/MS spectra and subsequently their structural similarities. In an MN, MS/MS spectra are compared pairwise based on spectral alignment to determine a correlation index cosine score assigned between each pair of spectra [9]. The more similar the MS/MS spectra are, the closer to one the cosine score is, and the stronger they are connected in a cluster. MN building is performed using online platforms such as GNPS or MetGem software [7,10]. The resulting MN considerably facilitates and increases the reliability of compound identification based on the use of standards and online database querying [11].

LC-HRMS/MS untargeted metabolomic analyses using MNs have been successfully applied in clinical toxicology to identify toxicants and their metabolites, including new psychoactive substances (NPS), belonging to the xenometabolome [12,13,14,15,16,17]. Indeed, in clinical toxicology laboratories, the identification of exogenous metabolites is based on the comparison of the MS data to a list of known compounds using a targeted approach. However, this approach limits the identification of metabolites to those contained in the used database and is therefore not suitable for the identification of new metabolites. In contrast, untargeted approaches with dedicated data processing allows for the identification of the involved toxicants and their metabolites in a comprehensive manner, especially in poisoned patients [14]. Identification of previously undescribed xenometabolites of commonly used drugs has been successfully performed using MNs [18]. Using this approach, new metabolites of quetiapine have been identified both from in vitro and human samples [18]. The usefulness of MNs in the identification of toxicants and their metabolites, namely the xenometabolome, was thus recently questioned [14,18]. Nevertheless, the reliability of MNs in the identification of the metabolites of a given xenobiotic as exhaustively as possible remains to be established.

The aim of our study was to develop a useful untargeted analytical workflow to extensively identify the toxicants and their metabolites in poisoned patients. We applied the proposed workflow to comprehensively identify metabolites of tramadol, a widely prescribed central-acting analgesic. Based on data obtained using LC-HRMS/MS with polarity switching in DDA mode, we first studied the analytical features of tramadol and its demethylated metabolites, i.e., O-desmethyl tramadol, N-desmethyl tramadol, and N,O-desmethyl tramadol [19,20]. We then analyzed urine and plasma samples obtained from a severely tramadol-poisoned 32-year-old female managed in the intensive care unit two days after self-ingestion of 9 g tramadol in a suicide attempt [21]. Plasma tramadol concentration on admission was 10.05 mg/L, a highly toxic concentration (toxic range, >1 mg/L) [19,20]. Despite optimal management, poisoning resulted in the patient’s death. To identify tramadol metabolites in an untargeted way, LC-HRMS/MS data corresponding to the urine and plasma extracts in this patient were used to build a MN in both positive and negative ion modes. This approach allowed for the identification of numerous additional phase I/II metabolites, including six previously unreported metabolites.

## 2. Results

### 2.1. Analytical Features and Fragmentation Patterns of Tramadol and Its Demethylated Metabolites

**Figure 1:**
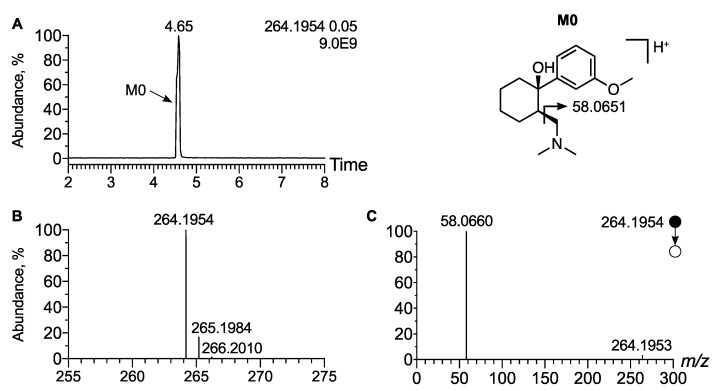
**Analytical features of tramadol.** (**A**) Extracted ion chromatogram of urine extract at *m*/*z* 264.1954 corresponding to [M + H]^+^ ion of tramadol. (**B**) Mass spectra of tramadol and its corresponding isotopic pattern. (**C**) MS/MS spectra of [M + H]^+^ ion of tramadol. Inspection of the MS/MS spectra allow for the identification of an intense peak at *m*/*z* 58.0660 corresponding to a methyl aziridium ion, indicative of the dimethyl amino radical of tramadol.

In order to establish the analytical behavior of tramadol and its demethyl metabo-lites, i.e., O-desmethyl tramadol, N-desmethyl tramadol, and N,O-didesmethyl tramadol, we first spiked a urine sample with certified analytical standards at 1 µg/mL and analyzed the sample using an LC-HRMS/MS system. In LC-HRMS, tramadol exhibited a peak at t_R_ = 4.65 min at *m*/*z* 264.1958 (Δppm < 1), corresponding to the [M + H]^+^ ion (Figure 1A). Moreover, the isotopic pattern was in accordance with the molecular formula of tramadol, i.e., C_16_H_25_NO_2_ (Figure 1B). The higher-energy C-trap Dissociation (HCD) MS/MS spectra exhibited an intense peak at *m*/*z* 58.0660 corresponding to a methyl aziridinium ion, diagnostic of the dimethyl amino radical of tramadol (Figure 1C). Moreover, a tiny peak at *m*/*z* 246.1851 corresponding to the loss of water (neutral loss of 18.0104, Δppm = 5) was detected. Based on these analytical features, native tramadol was unambiguously detected in the urine extract of the patient.

**Figure 2:**
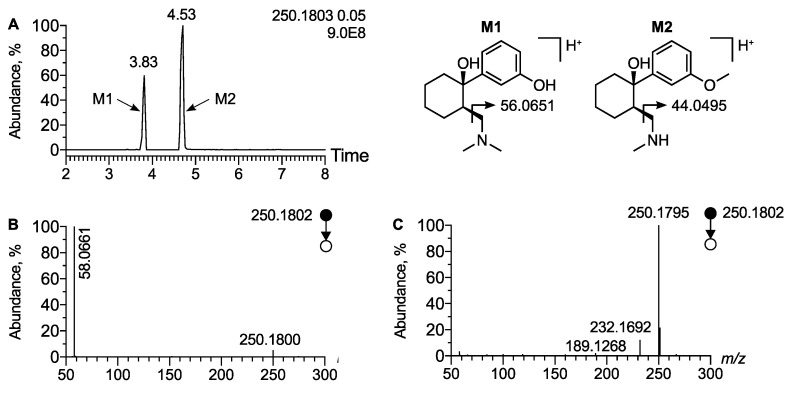
**Analytical features of the major phase I metabolites of tramadol.** (**A**) Extracted ion chromatogram of urine extract at *m*/*z* 250.1802 corresponding to [M + H]^+^ ion of desmethyl tramadol. MS/MS spectra of [M + H]^+^ ion of (**B**) O-desmethyl tramadol and (**C**) N-desmethyl tramadol.

We then assessed the analytical behavior of the widely described demethylated isomeric metabolites of tramadol, O-desmethyl tramadol (M1) and N-desmethyl tramadol (M2). The extracted ion chromatogram at *m*/*z* 250.1802 corresponding to the [M + H]^+^ ion of desmethyl tramadol displayed two peaks at t_R_ = 3.83 min and t_R_ = 4.53 min (Figure 2A). Inspection of corresponding MS/MS spectra allowed for the identification of the peak at t_R_ = 3.83 min as metabolite M1 and the peak at t_R_ = 4.53 min as metabolite M2. The MS/MS spectra of the first peak displayed a peak at *m*/*z* 58.0661 corresponding to the methyl-aziridinium ion, diagnostic of the dimethyl amino radical, i.e., the M1 metabolite (Figure 2B). In contrast, on the MS/MS spectra of M2 the expected aziridinium ion at *m*/*z* 44.0494 was not displayed, since the MS/MS was acquired between *m*/*z* 50 and *m*/*z* 1000 (Figure 2C). Regarding the analysis of the urine extract of the patient, we have identified tramadol, M1, M2, as well as two other known metabolites detected at *m/z* 222.1489 (Δppm = 1.8) and t_R_ = 3.41 min and at *m*/*z* 236.1645 (Δppm = 2.5) and t_R_ = 3.66 min, respectively (Supplementary Materials Figure S1). They were ascribed to N,N,O-desmethyl tramadol (M4) and N,O-desmethyl tramadol (M5), respectively, since no peak at *m*/*z* 58.0660 was displayed on MS/MS spectra, the same as for M2.

### 2.2. Molecular Network Built from Data Acquired Using LC-HRMS/MS in Positive Ion Mode

**Figure 3:**
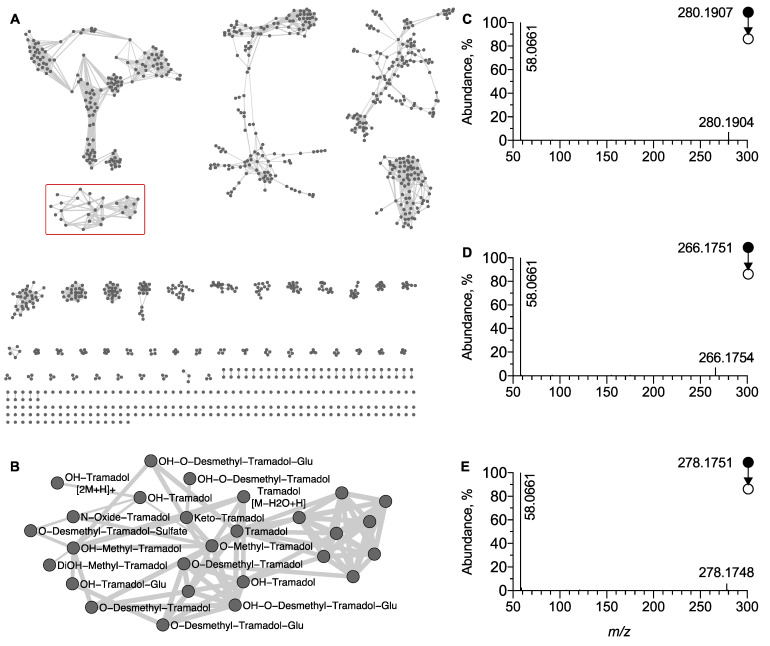
**Molecular network generated under positive ion mode.** (**A**) Global overview and (**B**) focus on the cluster of tramadol-related metabolites (C1 displayed as redbox). MS/MS spectra of [M + H]^+^ ion corresponding to (**C**) OH-tramadol, (**D**) O-desmethyl tramadol, and (**E**) keto tramadol, respectively. The peak at *m*/*z* 58.0660 displayed on each MS/MS spectra facilitated the clustering in the MN.

To identify additional tramadol metabolites in an untargeted manner, LC-HRMS/MS data acquired in positive ion mode were used to generate a MN. An overview of the MN is displayed in Figure 3. On this MN, the node corresponding to the MS/MS spectra of the [M + H]^+^ ion of tramadol was located and allowed to delimit the cluster (**C1**) of structurally related tramadol compounds, i.e., its metabolites. (Figure 3B). The fact that the node corresponding to M1 was included in this cluster is indicative that networking is based on the product ion at *m*/*z* 58.0660 and was supported by the lack of a node corresponding to M2, M4, and M5 (Figure 3B). The systematic annotation of the different nodes of the tramadol cluster in accordance to mass spectrometry fragmentation rules associated to tramadol, allowed for the identification of 15 phase I/II metabolites.

Among phase I metabolites, oxidative tramadol derivatives were detected as an [M + H]^+^ ion and identified as OH-tramadol (M6), OH-O-desmethyl tramadol (M32), or keto tramadol (M9), at *m*/*z* 280.1907 (Δppm = 0.7) and t_R_ = 3.60 min, at *m/z* 266.1751 (Δppm = 0.4) and t_R_ = 3.10 min and at *m*/*z* 278.1751 (Δppm = 0.7) and t_R_ = 3.7 min, respectively. Their MS/MS spectra all shared common product ions, i.e., the methyl aziridinium ion at *m/z* 58.0660, allowing their inclusion in cluster C1 (Figure 3C–E). It is noteworthy that in C1 several nodes were attributed to metabolite structural isomers of OH-tramadol since hydroxylation may occur on positions two, four, five, and six of the aromatic ring but also on the cyclohexyl radical. However, although these hydroxy metabolites exhibit the same *m*/*z* value and the same MS/MS pattern, they do not display the same retention time, leading to multiple nodes for metabolite structural isomers (Figure 3B and Figure S2).

The C1 cluster also showed nodes corresponding to phase II metabolites, including glucurono- and sulfo-conjugated metabolites. O-desmethyl tramadol glucuronide (M13), OH-tramadol glucuronide (M16) and OH-O-desmethyl tramadol glucuronide were detected in urine extracts as an [M + H]^+^ ion at *m*/*z* 426.2112 (Δppm = 0.2) and t_R_ = 3.28 min at *m*/*z* 456.2228 (Δppm = 3.7) and t_R_ = 3.62 min and at *m*/*z* 442.2075 (Δppm = 3.6) and t_R_ = 3.22 min, respectively. They were both included in cluster C1, since their MS/MS spectra display peaks corresponding to a methyl aziridinium ion (Figure 3B). It is noteworthy that the t_R_ values of the glucurono-conjugated metabolites were slightly lower than the corresponding non-conjugated metabolites in accordance with enhanced polarity due to the addition of the glucuronide radical. Finally, O-desmethyl tramadol sulfate (M20) was detected as an [M + H]^+^ ion at *m*/*z* 330.1372 (Δppm = 0.6) and t_R_ = 3.79 min in the urine sample. Similarly to the other metabolites, M20 also displayed a peak at *m*/*z* 58.0660, diagnostic of the dimethylamino radical and was included in cluster C1 (Figure 3B).

**Figure 4:**
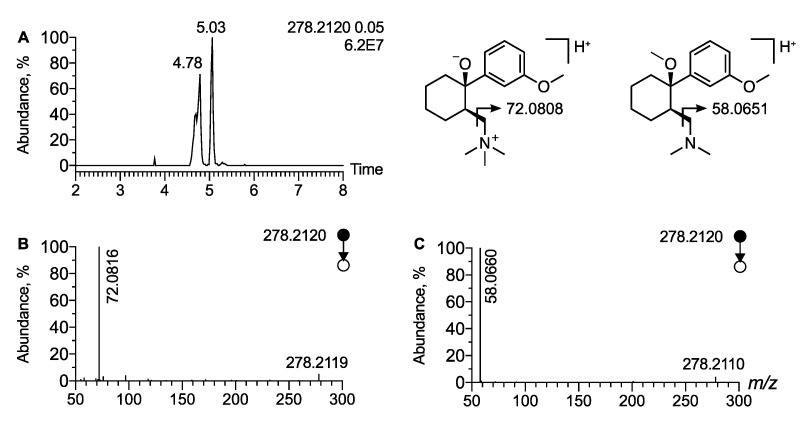
**Analytical features of methylated tramadol.** (**A**) Extracted ion chromatogram of urine extract at *m*/*z* 278.2120 corresponding to [M + H]^+^ ion of methyl tramadol. MS/MS spectra of [M + H]^+^ ion of (**B**) N-methyl tramadol and (**C**) O-methyl tramadol. The product ion at *m*/*z* 72.0816 correspond to a dimethylaziridium ion and allow for the annotation of N-methyl tramadol.

In cluster C1, a node corresponding to a precursor ion at *m*/*z* 278.2120 (Δppm = 2.2) is linked to tramadol. It corresponds to a mass shift of 14.0156 (Δppm = 3.6) and may be indicative of an additional methyl radical located on a hydroxyl or on the dimethyl amino radical (Figure 3B). Moreover, the isotopic pattern allowed the proposal of a molecular formula of C_17_H_27_NO_2_, strongly supporting that the ion at *m*/*z* 278.2120 corresponds to an [M + H]^+^ ion of methyl tramadol. The EIC at *m*/*z* 278.2120 displayed two peaks at t_R_ = 4.78 min and t_R_ = 5.03 min (Figure 4A). The MS/MS spectra of the peak at t_R_ = 5.03 min display the peak characteristic of methyl aziridinium ion at *m*/*z* 58.0660 (Δppm = 12) and was thus assigned to O-methylated tramadol (Figure 4C). In contrast, the MS/MS spectra of the peak at t_R_ = 4.78 min displayed no peaks at *m*/*z* 58.0655. However, an intense peak corresponding to a dimethyl aziridinium ion was observed at *m*/*z* 72.0815 (Δppm = 10) (Figure 4B). Therefore, the peak detected at *m*/*z* 278.2115 and t_R_ = 4.78 min was attributed to the [M + H]^+^ ion of N-methyl tramadol. It is noteworthy that N-methyl tramadol was eluted before O-methyl-tramadol as expected and that the two methyl tramadols have a t_R_ value slightly higher than tramadol (t_R_ = 4.65 min). Finally, cluster C1 also included two nodes corresponding to two additional methyl tramadol-derived metabolites. A peak at t_R_ = 3.5 min and *m*/*z* 294.2063 and another at t_R_ = 3.4 min and *m*/*z* 310.2013 were assigned as OH-O-methyl tramadol and DiOH-O-methyl tramadol, respectively, based on the same diagnostic product ion, i.e., *m*/*z* 58.0660 (Figure 3B). To the best of our knowledge, these four tramadol metabolites have not been previously reported.

Depending on which metabolic reaction occurred first, namely O- or N-desmethylation, two different networks were generated. The first one, which is mainly based on methyl aziridium product ions, led to C1 selectively including the O-desmethyl metabolites of tramadol. In contrast, the second network, generated thanks to data acquired at a 17.5 eV collision energy, included N-desmethyl metabolites of tramadol and is mainly based on the common loss of water and a product ion at *m*/*z* 189.1274 corresponding to the tramadol backbone (Supplementary Figure S3).

### 2.3. Molecular Network Built on Data Acquired Using LC-HRMS/MS in Negative Ion Mode

**Figure 5:**
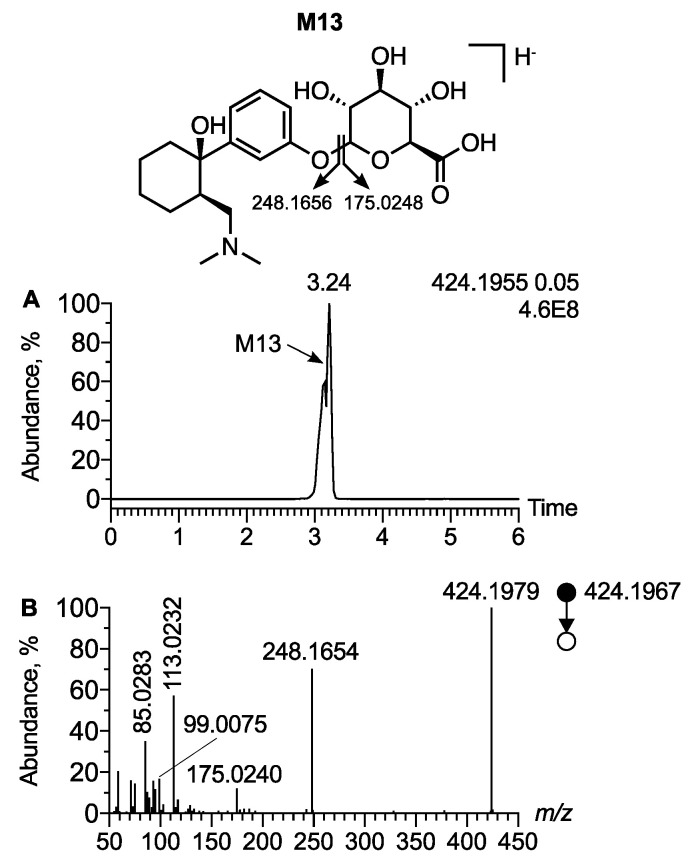
**Analytical features of O-desmethyl tramadol glucuronide in negative ion mode.** (**A**) Extracted ion chromatogram of urine extract at *m*/*z* 424.1955 corresponding to the [M − H]^−^ ion of desmethyl tramadol glucuronide. (**B**) MS/MS spectra of [M − H]^−^ ion of desmethyl tramadol.

The polarity switching ionization mode allowed for the acquisition of data in both positive and negative ion modes. While tramadol and its phase I metabolites were not detected in negative ion mode, the phase II metabolites, glucuro- and sulfo-conjugated metabolites, were detected. A peak at t_R_ = 3.24 min and *m*/*z* 424.1955 (Δppm = 2.8) was attributed as the [M − H]^−^ ion of O-desmethyl tramadol glucuronide (M13) (Figure 5A,B). The MS/MS spectra of O-desmethyl tramadol glucuronide displayed a peak at *m*/*z* 248.1654 resulting from a neutral loss of 176.0329, corresponding to the glucuronide radical (Figure 5C). The MS/MS spectra also displayed a peak at *m*/*z* 175.0240 corresponding to a trihydroxydihydropyrane carboxylate ion, characteristic of the glucuronide radical (Figure 5C). MS/MS spectra displayed other product ions corresponding to a subsequent fragmentation of trihydroxydihydropyrane carboxylate ion, supporting the presence of the glucuronide. Besides trihydroxydihydropyrane carboxylate, the use of SIRIUS software allowed for the identification of the main product ions characteristic of the glucuronide radical: dihydrofurane carboxylate, oxobutanoate, hydroxyacrylate, and cycloprane carboxylate, detected at *m*/*z* 113.0232, *m*/*z* 99.0075, *m*/*z* 87.0076, and *m*/*z* 85.0283, respectively.

**Figure 6:**
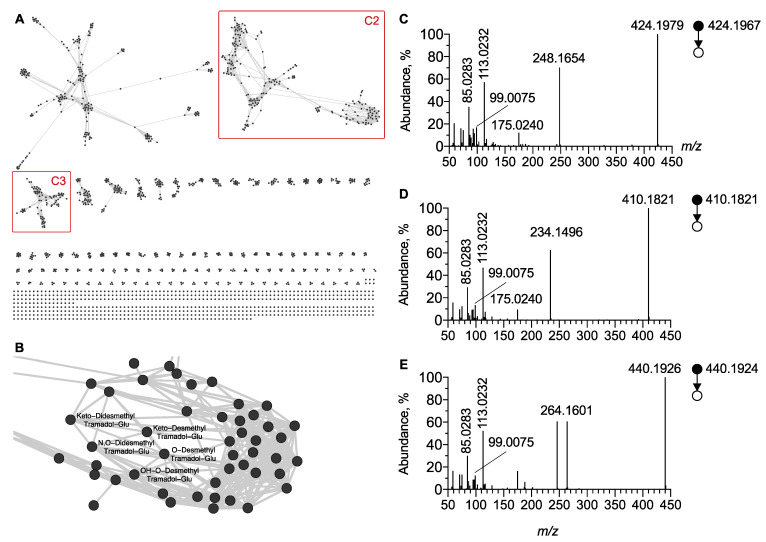
**Molecular network generated under negative ion mode.** (**A**) Global overview and (**B**) focus on the C2 cluster of glucurono-conjugated molecules including tramadol. MS/MS spectra of [M − H]^−^ ion corresponding to (**C**) O-desmethyl tramadol glucuronide, (**D**) N,O-didesmethyl tramadol glucuronide and (**E**) OH-O-desmethyl tramadol glucuronide, respectively. Note that unidentified nodes correspond to glucurono-conjugated endogenous metabolites.

Based on the MS/MS spectra obtained using negative ion mode and displaying these product ions, a cluster (**C2**) was generated. C2 included all glucurono-conjugated tramadol metabolites (Figure 6). However, based on glucuronide radical fragmentation, C2 also included glucurono-conjugated metabolites of other xenobiotics. The careful inspection of the C2 cluster allowed for the identification of three glucurono-conjugated tramadol metabolites including O-desmethyl tramadol glucuronide (M13), N,O-desmethyl tramadol glucuronide (M15) and OH-O-desmethyl tramadol glucuronide (M17) (Figure 6C–E). Notably, since C2 included M13, M15, and M17 in negative ion mode, the networking did not depend on the structural specificity of the phase I tramadol metabolites, only on that from the glucuronide part. In contrast, in positive ion mode M13 and M17 were included in the corresponding cluster C1, as networking is based on the dimethylamino radical. Instead, M15 was not included in C1 as it contains a methylamino radical. Furthermore, inspection of the MN generated in negative ion mode was useful to identify two previously undescribed phase II metabolites, namely desmethyl keto tramadol N-glucuronide and didesmethyl keto tramadol N-glucuronide.

**Figure 7:**
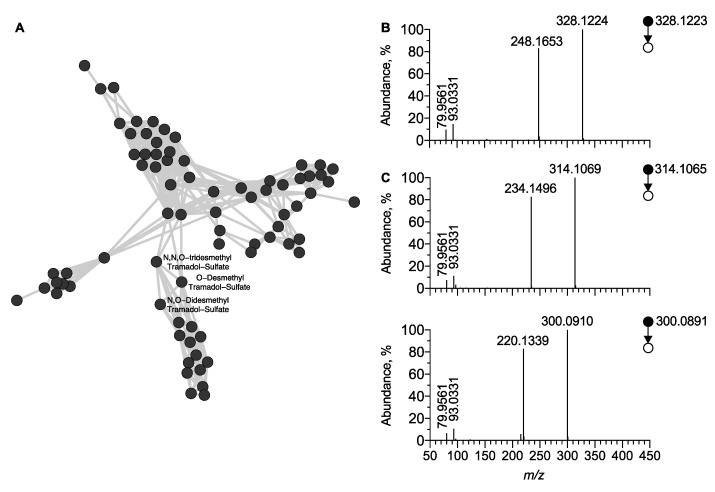
**Molecular network of sulfo-conjugated molecules generated under negative ion mode.** (**A**) Overview on the C3 cluster of sulfo-conjugated molecules including tramadol-related metabolites. MS/MS spectra of [M − H]^−^ ion corresponding to (**B**) O-desmethyl tramadol sulfate, (**C**) N,O-didesmethyl tramadolsulfate, and N,N,O-desmethyl tramadol sulfate, respectively. Note that unidentified nodes correspond to sulfo-conjugated endogenous metabolites.

Similarly, a third cluster (**C3**), part of the MN generated in negative ion mode, was attributed to sulfo-conjugated molecules (Figure 6A and Figure 7A). The [M − H]^−^ ion of *O*-desmethyl tramadol sulfate was detected at t_R_ = 3.83 min and *m*/*z* 328.1207 (Δppm = 1.8) and its MS/MS spectra displayed a peak at m/z 248.1653 (Δppm = 1.8). This resulted from the neutral loss of 79.9568 Th corresponding to the sulfate radical (Figure 7B). The MS/MS spectra also displayed a peak at *m*/*z* 79.9561 corresponding to the dioxosulfanolate ion, characteristic of the sulfate radical. Using the same approach, three additional sulfo-conjugated tramadol metabolites were identified from the cluster C3, including O-desmethyl tramadol sulfate (M20), N,O-desmethyl tramadol sulfate (M22), and N,N,O-desmethyl tramadol sulfate (M21) (Figure 7B–D).

### 2.4. Summary of Identified Tramadol Metabolites

Table 1 shows all tramadol metabolites identified in this study and includes the main analytical features, i.e., t_R_ and *m*/*z* values of precursor ions. The MNs generated in positive and negative ion modes allowed identifying 25 tramadol metabolites. For each metabolite, the level of metabolite identification is reported in Supplementary Table S1 according to the metabolomics standards initiative guidelines [22]. Among them, ten were phase I and 15 were phase II tramadol metabolites. The phase I tramadol metabolites included hydroxylated, demethylated, carbonylated, or N-oxide compounds. The phase II tramadol metabolites involved glucurono- or sulfo-conjugated molecules as well as methylated compounds. These 25 metabolites were identified in the urine of the patient at five sampling times (Table 1). Several of the identified tramadol metabolites in urine were also detected in the plasma.

In this study, metabolites of tramadol were detected in a fatal intoxication case of tramadol. To determine whether the reported metabolites of tramadol were detected in plasma and urine samples in additional patients, we performed a qualitative and quantitative study in four patients who have ingested tramadol. Plasma tramadol concentrations ranged from 0.49 to 0.005 mg/L, indicating that all of the investigated patients were in therapeutic range. The main characteristics of the titration curve used for quantitative determination of tramadol, O-desmethyl tramadol and N-desmethyl tramadol are displayed in supplementary Table S2. A dose-dependent correlation was observed for the metabolites detected. Indeed, for patient number two, for whom the tramadol concentration was determined to be 0.49 mg/L, we identified 15 metabolites of tramadol in urine and plasma of the 25 metabolites reported in this study (Supplementary Table S3). In contrast, for patient number three, for whom the concentration was determined to be 0.023 mg/L, we identified 12 metabolites of tramadol in plasma and 13 in the urine sample of the 25 reported metabolites (Supplementary Table S3).

## 3. Discussion

In the present study, we aimed to propose a convenient analytical workflow based on MNs to identify tramadol and its metabolites in urine and blood samples obtained in a fatal tramadol poisoning case. Tramadol is a widely prescribed analgesic with centrally acting μ-opioid and serotonin and norepinephrine reuptake inhibiting properties. Tramadol poisoning may also result in early seizures and cardiovascular disturbances [20]. CYP2D6-mediated O-demethylation of tramadol in the liver leads to the formation of O-desmethyl tramadol, an active metabolite with higher affinity for μ-opioid receptors compared to tramadol [19]. A comprehensive investigation of tramadol metabolites was thus required to identify all active compounds generated by the metabolism in vivo. For this purpose, tramadol metabolism has been previously investigated in healthy volunteers involving an intense hepatic biotransformation [19,24,25]. To the best of our knowledge, no comprehensive characterization of tramadol metabolites in a poisoned patient has been previously performed.

Our analytical procedure included a sample preparation with an extraction step of both hydrophilic and hydrophobic compounds using a mixture of solvents (isopropanol/acetonitrile/water, 3/3/2, *v*/*v*/*v*). Extracts were analyzed using LC-HRMS/MS in both positive and negative ion modes. UHPLC offers high-resolution chromatography, improving peak shape and therefore separation allowing to circumvent or at least limits, ion suppression. Together with the high-resolution mass spectrometer used, it allowed us to develop a highly sensitive analytical method suitable for performing an exhaustive identification of drug metabolites. The analytical system operated in a data-dependent acquisition mode allowing for the acquisition of tandem mass spectra on the three most intense ions per cycle. This analytical procedure allows a comprehensive detection of metabolites exhibiting a wide structural diversity and covers a large concentration range. To facilitate and ensure the reliable identification of tramadol metabolites, MS, MS/MS, and t_R_ data analyses were associated to the MN strategy, allowing for the identification of 25 phase I/II tramadol metabolites, including six metabolites that have not been previously described in humans, mammals, or in in vitro models of drug metabolism.

Among the 25 metabolites detected in the patient urine involved in this study, some have been already reported, such as phase I demethylated metabolites, i.e., O-desmethyl tramadol, N-desmethyl tramadol, and N,O-didesmethyl tramadol, widely described with routine quantitative methods. Regarding other phase I/II metabolites, Wu et al. have performed comprehensive studies reporting 26 tramadol metabolites in the urine of dogs, rats, and healthy volunteers [23,26]. Investigating metabolites in biological samples in poisoned patients is highly relevant in contrast to drug metabolism studies using both in vitro and in vivo models. In our study, 19 tramadol metabolites described in urine samples of healthy volunteers were fully characterized. In addition, our study is the first to report 23 tramadol metabolites in the plasma of a severely tramadol-poisoned patient. Among them, LC-HRMS/MS untargeted analysis associated to an MN allowed to describe six metabolites that have not been previously reported. It is noteworthy that the chemical structures proposed for these novel metabolites remain putative and that additional experiments would be required to unambiguously identify these novel metabolites. Some of these phase II metabolites corresponded to methylated tramadol derivatives. Methylation represents a minor phase II biotransformation pathway [27]. As no standard for these metabolites was commercially available, no quantification of methylated metabolites as compared to other metabolites was performed. Nevertheless, the pharmacodynamics of synthetized O-methyl tramadol have been already described, indicating a 20-fold higher norepinephrine reuptake inhibition than tramadol [28]. In contrast, no change in μ-opioid receptor affinity was observed with O-methyl tramadol, when compared to tramadol [28]. Since this was detected in the plasma, this metabolite could be considered as a therapeutic drug and for poisoning monitoring. Our results may be considered as a proof of concept demonstrating the effectiveness of a metabolomic analysis performed in an untargeted manner and associating an MN data processing strategy to perform a comprehensive identification of metabolites in poisoning. The proposed analytical workflow was used to describe the xenometabolome of tramadol in the particular case of a massive intoxication involving an unusual high concentration of one single drug. The fact that, in addition to common metabolites encountered in tramadol intoxication, unusual ones were detected and identified probably reflects the high analytical sensitivity of the analytical method and use of the molecular network approach. Moreover, massive intoxication with tramadol may lead to normal metabolic pathway saturation, triggering uncommon pathways which in turn result in the formation of unusual metabolites.

If the toxicant involved in exposure is known, this approach based on the exhaustive inspection of LC-HRMS/MS data may allow for the identification of unreported metabolites possibly helpful for clinical monitoring and prognostication. This approach is also useful to improve the knowledge of metabolism of a given molecule. Finally, it is also helpful for the determination of chemical structures such as those of new psychoactive substances, allowing for the characterization of the corresponding in vivo metabolites, a prior step in determining pharmacokinetic properties. The use of MNs is thus a valuable approach facilitating a comprehensive systematic identification without a priori knowledge of such unknown compounds and their metabolites.

The identification of metabolites using MNs in untargeted metabolomic dataset may be performed through different approaches. Identification may be achieved by database querying, a highly efficient method for the dereplication of described metabolites, allowing for the anchorage of the MN prior to the propagation of annotation. Otherwise, identification may be performed using commercial standard compounds, if available. Regardless of the chosen approach, in-depth knowledge of the rules governing fragmentation in mass spectrometry should be perfectly established for the optimal use of MN. In our hands, in positive ion mode, HCD MS/MS spectra of tramadol mainly exhibited an intense peak at *m*/*z* 58.0660, corresponding to methyl aziridium ion characteristic of dimethyl amino radical. This approach allowed the creation of a cluster including tramadol and its metabolites containing the dimethyl amino radical but not N-demethylated metabolites. In negative ion mode, glucurono-conjugated and sulfo-conjugated metabolites were included in two different MN clusters. HCD MS/MS spectra of glucuro- and sulfo-conjugated metabolites mainly displayed product ions or neutral loss related to glucuronide or sulfate, respectively. In other words, the molecular network generated from the data corresponding to LC-HRMS/MS urine extract analysis displayed a series of clusters originating in endogenous and exogenous metabolites networking. In the case of a patient poisoned by tramadol, one of these cluster links tramadol as well as several of its metabolites. On one hand, a cluster may only include metabolites of tramadol as shown in the cluster C1; on the other hand, an additional cluster included tramadol-related metabolites in addition to an endogenous one. It is especially the case for C2 and C3 which network sulfo- and glucurono-conjugates of tramadol metabolites as well as endogenous metabolites. In conclusion, MS fragmentation rules are key to perform a comprehensive identification of the metabolites displayed as nodes in an MN.

## 4. Materials and Methods

**Chemicals and Reagents**. Water, acetonitrile, isopropanol, and methanol of LC-MS grade were obtained from Sigma-Aldrich (Saint-Quentin-Fallavier). Tramadol, O-desmethyl tramadol, N-desmethyl tramadol, and N,O-didesmethyl tramadol were purchased from LoGiCal^®^ (LGC GmBH, Luckenwalde, Germany). Tramadol C13, D3 (1 mg/mL as free base in methanol, LGC) was used as an internal standard.

**Urine and plasma sampling**. This laboratory investigation, included in a clinical study on the pharmacokinetic/pharmacodynamic relationships of psychotropic drugs, was conducted according to the Helsinki principles, declared to the Commission Nationale de l’Informatique et des Libertés (declaration number, 2067659), and approved by the ethics committee of the French Society of Intensive Care (protocol number, FICS20020231). Due to the non-interventional design of the study, written consent was waived. Since the patient was unconscious during her management in the intensive care unit before her death, her next of kin was fully informed why the research was being conducted and how the data will be used. All of the urine and plasma samples were collected in the toxicological critical care of the university hospital.

The case involved a Caucasian 32-year-old women who had ingested 9000 mg sustained-release tramadol [21]. The patient was found with hypoglycemia, bradypnea, and hypotension [21]. The electroencephalogram displayed a non-reactive encephalopathy. Despite optimal management in the intensive care unit, brain death occurred on day 12. Regarding the plasma and the urine samples analyzed in this study, they were collected at day 2, day 3, and day 11 following the tramadol ingestion. A heparin tube was used for plasma collection and all of the samples were stored to 4 °C prior to the analyses.

**Sample preparation**. A volume of 350 µL of acetonitrile and 350 µL of isopropanol was added to 250 µL of urine samples. The precipitated proteins were pelleted by centrifugation at 14,000 rpm for 10 min. Supernatants were collected and solvents were evaporated at 40 °C under a gentle steam of nitrogen. Dry residues were resuspended in water/acetonitrile/methanol (50:25:25 *v*/*v*/*v*) and subsequently, 10 µL of the extracts were injected into the analytical system.

**Analytical conditions**. The LC-HRMS/MS system was based on a Vanquish^®^ LC pump and autosampler coupled to a QExactive Focus^®^ mass spectrometer equipped with a heated electrospray ionization (HESI) probe operating in positive and negative ion modes. The analytical system was managed using TraceFinder^®^ 4.0 software (ThermoFisher Scientific, Bremen, Germany). Liquid chromatography was performed on an Accucore^®^ Phenyl Hexyl (100 × 2.1 mm, 2.6 µm, (ThermoFisher Scientific, Bremen, Germany)) column maintained at 40 °C. The flow rate was set at 500 µL/min. A binary gradient system was used for the elution and consisted of water with 2 mmol/L ammonium formate as solvent A and methanol/acetonitrile mixture with 2 mmol/L ammonium formate (50:50) as solvent B. Both solvents A and B contained 0.1% formic acid. The eluent was maintained at 1% B for 1 min, increased to 99% B in 10 min, held at 99% B for 1.5 min before returning to 1% B, and finally, held for 4 min. The mass spectrometer parameters were as follows: ionization voltage was 3.0 kV for positive ion mode and 2.2 kV for negative ion mode; sheath gas and auxiliary gas were 35 and 15 arbitrary units, respectively; S-lens RF level 60; and vaporizer temperature and capillary temperature were both set at 320 °C. Nitrogen was used for spray stabilization, for collision induced dissociation experiments in the HCD cell, and as the damping gas in the C-trap. AGC target was fixed to 1E5 for MS/MS experiments. Transient time was fixed to 120 and 50 ms for full MS and MS/MS scans, respectively. The mass spectrometer was calibrated in the positive and negative modes prior to analyses. Data were acquired in a data-dependent MS2 (ddMS2) mode. In this mode, a full-scan acquisition was performed at a resolution of 70,000 between *m*/*z* 100 to 1000, followed by the acquisition of MS/MS spectra of the three most intense ions, at a resolution of 17,500 with an isolation window of 2 Th. The acquisition of MS and MS/MS data was managed by Thermo Scientific TraceFinder^®^ software.

**Data processing parameters**. Raw data files acquired in positive and negative ion modes were converted into open source mzXML files under MSConvert 3.0™. The data processing was performed under MZmine 2.53 [29]. Briefly, MS and MS/MS spectral data were extracted using a mass detection noise level set at 1E5 and 5E3, respectively. Extracted ion chromatograms were then systematically built using the ADAP algorithms (minimum group size of 4 scans, a group intensity threshold of 500,000, and an *m*/*z* tolerance of 10 ppm) [30]. The ADAP wavelets chromatogram deconvolution algorithm was then applied and set at the following parameters: signal to noise ratio = 0, coefficient/area ratio = 20, peak duration range = 0.05–3 min, and retention time wavelet range = 0.0–0.15. Deisotope chromatograms were grouped using the isotopic peaks grouper algorithm set at *m*/*z* and t_R_ tolerances of 10 ppm and 0.1 min, respectively. Peak alignment of samples was then achieved using the join aligner method with parameters set at *m*/*z* and t_R_ tolerances of 10 ppm and 0.15 min, respectively. MS/MS scans were associated to the corresponding MS scans using a *m*/*z* and t_R_ tolerance of 10 ppm and 0.15 min, respectively. The gap-filling process was performed on the peak list using the same RT and *m*/*z* range gap filler module with a *m*/*z* tolerance of 10 ppm.

**Molecular Network generation**. The MNs were generated using the feature-based molecular networking workflow of the Global Natural Products Social platform and MetGem software [9,10]. MNs were built using LC-MS/MS data obtained from both the urine and plasma samples analyses. The following settings were used to build the network: minimum pairs Cos > 0.60, parent ion mass tolerance = 0.02 Da, fragment ion mass tolerance = 0.02, network topK < 150, minimum matched peaks = 3, and minimum cluster size = 2. The library spectra inquiries were performed using the same parameter values as those defined for the network building. The MNs were finally visualized and annotated using Cytoscape 3.4.0™ software (San Diego, CA, USA) [31].

**Identification of tramadol metabolites**. The structure assignment of tramadol and its metabolites was based on MS and MS/MS data, using a tolerance window of 5 and 15 ppm, respectively. Identification was supported by experimental chromatographic retention time (t_R_) values. SIRIUS 4.0 was used to support molecular formula determination and metabolite identification [32].

## 5. Conclusions

The present study demonstrates the usefulness of MN to process data obtained with an untargeted metabolomic analysis using liquid chromatography coupled to high-resolution tandem mass spectrometry to comprehensively identify tramadol and its related metabolites in biological samples in a tramadol-poisoned patient. The analytical workflow we proposed can be applied to many types of toxicants to improve the knowledge of drug metabolism and identify undescribed metabolites. This approach is suitable to highlight biomarkers as possible prognosticators. The newly identified metabolites need to be tested in additional samples to validate if their monitoring based on a quantitative approach contributes to poisoned patient management. 

## Figures and Tables

**Table 1:** Analytical features of tramadol and its metabolites detected in plasma or urine of the intoxicated patient.

Name	Metabolites	Plasma	Urine	ESI+		ESI−		Reference
				*m*/*z* (∆ppm)	t_R_	*m/z* (∆ppm)	t_R_	
M0	Tramadol	Detected	Detected	264.1963 (2)	4.65	/	/	[23]
M1	O-desmethyl tramadol	Detected	Detected	250.1802 (0)	3.83	/	/	[23]
M2	N-desmethyl tramadol	Detected	Detected	250.1802 (0)	4.59	/	/	[23]
M3	N,N-desmethyl tramadol	Detected	Detected	236.1639 (−3)	3.9	/	/	[23]
M4	N,N,O-desmethyl tramadol	Not detected	Detected	222.1485 (−2)	3.41	/	/	[23]
M5	N,O-desmethyl tramadol	Detected	Detected	236.1639 (−3)	3.66	/	/	[23]
M6	OH-tramadol	Detected	Detected	280.1905 (−1)	3.7	/	/	[23]
M7	OH-N-desmethyl tramadol	Detected	Detected	266.1752 (0)	3.62	/	/	[23]
M8	OH-didesmethyl tramadol	Detected	Detected	252.1591 (2)	3.2	/	/	[23]
M9	Keto tramadol	Detected	Detected	278.1749 (−1)	3.7	/	/	[23]
M13	O-desmethyl tramadol glucuronide	Detected	Detected	426.2113 (0)	3.28	424.1967 (3)	3.24	[23]
M15	N,O-desmethyl tramadol glucuronide	Detected	Detected	412.1966 (0)	3.29	410.1802 (−2)	3.3	[23]
M16	OH-tramadol glucuronide	Detected	Detected	456.2211 (−4)	3.62	454.2084 (3)	3.62	[23]
M17	OH-O-desmethyl tramadol glucuronide	Detected	Detected	442.2056 (−4)	3.22	440.1915 (0)	3.22	[23]
M18	OH-N,N-desmethyl tramadol glucuronide	Not detected	Detected	428.1913 (0)	3.23	/	/	[23]
M20	O-desmethyl tramadol sulfate	Detected	Detected	330.1372 (1)	3.79	328.1207 (−2)	3.83	[23]
M21	N,N,O-desmethyl tramadol sulfate	Detected	Detected	302.1055 (−1)	3.86	300.0902 (1)	3.83	[23]
M22	N,O-desmethyl tramadol sulfate	Detected	Detected	316.1217 (1)	3.94	314.1065 (3)	3.92	[23]
M31	N-oxide tramadol tramadol	Detected	Detected	280.1906 (−2)	4.76	/	/	[23]
M32	OH-O-desmethyl tramadol	Detected	Detected	266.1752 (0)	3.10	/	/	[23]
M37	Desmethyl keto tramadol N-glucuronide	Detected	Detected	/	/	438.1749 (−5)	4.6	Not reported
M38	Didesmethyl keto tramadol N-glucuronide	Detected	Detected	/	/	424.1623 (2)	4.3	Not reported
M33	O-Methyl Tramadol	Detected	Detected	278.212 (2)	5.03	/	/	Not reported
M34	N-Methyl Tramadol	Detected	Detected	278.2121 (3)	4.78	/	/	Not reported
M35	OH-Methyl Tramadol	Detected	Detected	294.2063 (0)	3.4	/	/	Not reported
M36	DiOH-Methyl Tramadol	Detected	Detected	310.2016 (1)	3.5	/	/	Not reported

## Data Availability

Raw data files are available in the open-source database MetaboLights project MTBLS5366 at www.ebi.ac.uk/metabolights/MTBLS5366 (accessed on 23 June 2022).

## Supplementary Materials

The following supporting information can be downloaded at: https://www.mdpi.com/article/10.3390/metabo12070665/s1, Figure S1: Analytical features of desmethyl metabolites of tramadol; Figure S2: Isomers of OH-Tramadol; Figure S3: Focus on the molecular network of N-demethylated metabolites of tramadol; Table S1: Level of metabolite identification of each annotated metabolites according to the metabolomics standards initiative guidelines.; Table S2: Limit of quantification, linear dynamic range, linearity of the plot of area response ratio versus concentration, correlation coefficient for tramadol, N-desmethyl-tramadol and O-desmethyl-tramadol.
