# Supplementary material for: Molecular Network-Based Identification of Tramadol Metabolites in a Fatal Tramadol Poisoning
Source: Metabolites. 2022 Jul 19;12(7):665. doi: 10.3390/metabo12070665 (PMC9323855; doi:10.3390/metabo12070665)
Supplement: Supplementary file 1 [file metabolites-12-00665-s001.zip › metabolites-1808943-supplementary.pdf]

Molecular Network-Based Identification of Tramadol Metabolites in a Fatal Tramadol Poisoning

Romain Magny[1,2,*], Nicolas Auzeil[2], Bertrand Lefrère[1], Bruno Mégarbane[3,4], Pascal Houzé[1,5] and Laurence Labat[1,4]

[1] Laboratoire de Toxicologie, Fédération de Toxicologie, AH-HP, Hôpital Lariboisière, Paris, France

[2] Université de Paris, CNRS, CiTCoM, Paris, France

[3] Réanimation Médicale et Toxicologique, Fédération de Toxicologie de l'AH-HP, Hôpital Lariboisière, Paris, France

[4] Inserm, UMRS-1144, Université Paris Cité, Paris, France

[5] Faculté de Sciences Pharmaceutiques Et Biologiques, Unité de Technologies Chimiques Et Biologiques Pour La Santé (UTCBS), Université Paris Cité, CNRS UMR8258, Inserm U1022, Paris, France.

[*]Corresponding authors: romain.magny@inserm.fr

Figure S1. Analytical features of desmethyl metabolites of tramadol. (A) Extracted ion chromatogram at *m/z* 236.1645 corresponding to N,O-desmethyl-tramadol and (B) extracted ion chromatogram at *m/z* 222.1489 corresponding to N,N,O-desmethyl-tramadol.

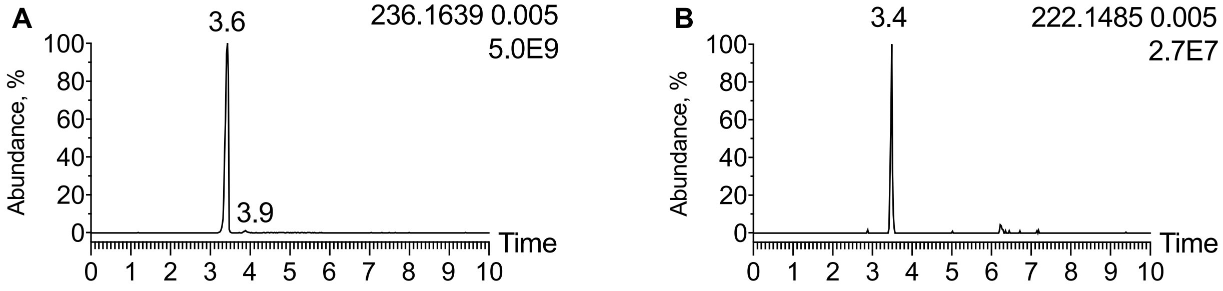

Figure S2. Isomers of OH-Tramadol. Extracted ion chromatogram at m/z 280.1905 corresponding to the $[M+H]^+$ ion of OH-tramadol. Note that the peak at t_R = 4,62 min correspond to the N-oxyde tramadol metabolite.

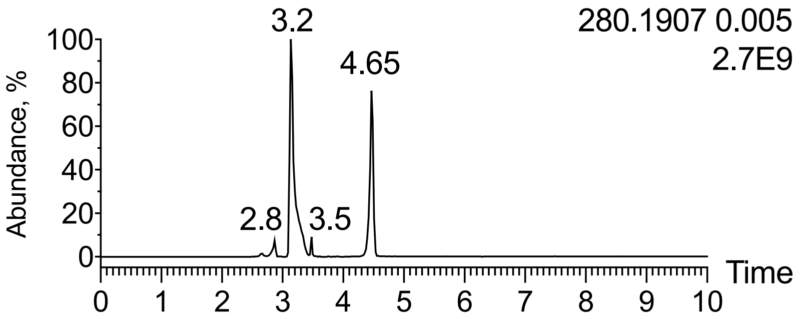

Figure S3. Focus on the molecular network of *N*-demethylated metabolites of tramadol generated using LC-HRMS/MS data acquired in positive ion mode at a 17.5 eV collision energy.

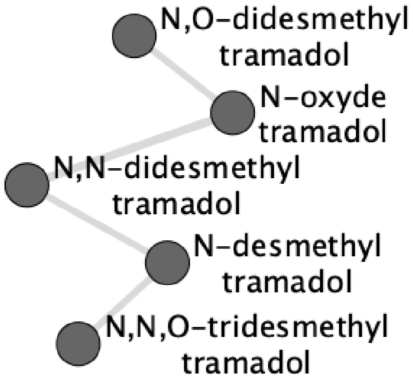

Table S1. Level of metabolite identification of each annotated metabolites according to the metabolomics standards initiative guidelines.

Metabolites	Identification level
Tramadol	1
O-Desmethyl-Tramadol	1
N-Desmethyl-Tramadol	1
N,N-Desmethyl-Tramadol	2
N,N,O-Desmethyl-Tramadol	2
N,O-Desmethyl-Tramadol	1
OH-Tramadol	2
OH-N-Desmethyl-Tramadol	2
OH-Didesmethyl-Tramadol	2
Keto-Tramadol	2
O-Desmethyl-Tramadol-Glucuronide	2
N,O-Desmethyl-Tramadol-Glucuronide	2
OH-Tramadol-Glucuronide	2
OH-O-Desmethyl-Tramadol-Glucuronide	2
OH-N,N-Desmethyl-Tramadol-Glucuronide	2
O-Desmethyl-Tramadol-Sulfate	2
N,N,O-Desmethyl-Tramadol-Sulfate	2
N,O-Desmethyl-Tramadol-Sulfate	2
N-oxide-Tramadol	2
OH-O-Desmethyl-Tramadol	2
Desmethyl-keto-tramadol-N-Glucuronide	2
Didesmethyl-keto-tramadol-N-Glucuronide	2
O-Methyl-Tramadol	2
N-Methyl-Tramadol	2
OH-Methyl-Tramadol	2
DiOH-Methyl-Tramadol	2

Table S2. Limit of quantification, linear dynamic range, linearity of the plot of area response ratio *versus* concentration, correlation coefficient for tramadol, N-desmethyl-tramadol and O-desmethyl-tramadol. Tramadol-C13, D3, HCl was used as internal standard.

Metabolites	LOQ (µg/L)	Linear dynamic range	Linear regression equation	Correlation coefficient (R^2)
Tramadol	2.5	400	$y = 0.01628x + 0.1132$	0.998
O-desmethyl-tramadol	2.5	400	$y = 0.00490x + 0.0025$	0.996
N-desmethyl-tramadol	2.5	400	$y = 0.01248x - 0.02008$	0.998

Table S3. Tramadol metabolites identified in the four investigated patients which have taken tramadol at therapeutic concentration. D.: detected, N.D.: not detected

	Patient #1		Patient #2		Patient #3		Patient #4	
	**Plasma**	**Urine**	**Plasma**	**Urine**	**Plasma**	**Urine**	**Plasma**	**Urine**
**Quantification**								
Tramadol	200	N.A.	490	N.A.	23	N.A.	5.1	N.A.
O-Desmethyl-Tramadol	91	N.A.	143	N.A.	7	N.A.	/	N.A.
N-Desmethyl-Tramadol	39	N.A.	37	N.A.	5	N.A.	/	N.A.
**Identification**								
Tramadol	D.	D.	D.	D.	D.	D.	D.	D.
O-Desmethyl-Tramadol	D.	D.	D.	D.	D.	D.	D.	D.
N-Desmethyl-Tramadol	D.	D.	D.	D.	D.	D.	D.	D.
N,N-Desmethyl-Tramadol	D.	D.	D.	D.	D.	D.	N.D.	N.D.
N,N,O-Desmethyl-Tramadol	D.	D.	N.D.	D.	N.D.	D.	N.D.	D.
N,O-Desmethyl-Tramadol	D.	D.	D.	D.	D.	D.	D.	D.
OH-Tramadol	D.	D.	D.	D.	D.	D.	D.	D.
OH-N-Desmethyl-Tramadol	N.D.	N.D.	N.D.	N.D.	N.D.	N.D.	N.D.	N.D.
OH-Didesmethyl-Tramadol	N.D.	N.D.	N.D.	N.D.	N.D.	N.D.	N.D.	N.D.
Keto-Tramadol	N.D.	N.D.	N.D.	N.D.	N.D.	N.D.	N.D.	N.D.
O-Desmethyl-Tramadol-Glucuronide	D.	D.	D.	D.	D.	D.	N.D.	D.
N,O-Desmethyl-Tramadol-Glucuronide	D.	D.	D.	D.	D.	D.	N.D.	N.D.
OH-Tramadol-Glucuronide	D.	D.	D.	D.	D.	D.	N.D.	N.D.
OH-O-Desmethyl-Tramadol-Glucuronide	D.	D.	D.	D.	D.	D.	N.D.	N.D.
OH-N,N-Desmethyl-Tramadol-Glucuronide	N.D.	N.D.	N.D.	N.D.	N.D.	N.D.	N.D.	N.D.
O-Desmethyl-Tramadol-Sulfate	D.	D.	D.	D.	D.	D.	N.D.	N.D.
N,N,O-Desmethyl-Tramadol-Sulfate	N.D.	N.D.	N.D.	N.D.	N.D.	N.D.	N.D.	N.D.
N,O-Desmethyl-Tramadol-Sulfate	D.	D.	D.	D.	D.	D.	N.D.	N.D.

	Patient #1		Patient #2		Patient #3		Patient #4	
	**Plasma**	**Urine**	**Plasma**	**Urine**	**Plasma**	**Urine**	**Plasma**	**Urine**
**Quantification**								
Tramadol	200	N.A.	490	N.A.	23	N.A.	5.1	N.A.
O-Desmethyl-Tramadol	91	N.A.	143	N.A.	7	N.A.	/	N.A.
N-Desmethyl-Tramadol	39	N.A.	37	N.A.	5	N.A.	/	N.A.
**Identification**								
N-oxide-Tramadol	D.	D.	D.	D.	D.	D.	N.D.	N.D.
OH-O-Desmethyl-Tramadol	N.D.	N.D.	N.D.	N.D.	N.D.	N.D.	N.D.	N.D.
Desmethyl-keto-tramadol-N-Glucuronide	D.	D.	D.	D.	N.D.	D.	N.D.	N.D.
Didesmethyl-keto-tramadol-N-Glucuronide	N.D.	N.D.	N.D.	N.D.	N.D.	N.D.	N.D.	N.D.
O-Methyl-Tramadol	D.	D.	D.	D.	N.D.	N.D.	N.D.	N.D.
N-Methyl-Tramadol	D.	D.	D.	D.	N.D.	N.D.	N.D.	N.D.
OH-Methyl-Tramadol	N.D.	N.D.	N.D.	N.D.	N.D.	N.D.	N.D.	N.D.
DiOH-Methyl-Tramadol	N.D.	N.D.	N.D.	N.D.	N.D.	N.D.	N.D.	N.D.
Total	16	16	15	16	12	14	4	6
